# Doxycycline-Induced Hand Tremors: Case Report and Review of Antibiotic-Associated Tremors

**DOI:** 10.7759/cureus.10782

**Published:** 2020-10-03

**Authors:** Joanne S Jacob, Philip R Cohen

**Affiliations:** 1 Internal Medicine, Baylor College of Medicine, Houston, USA; 2 Dermatology, San Diego Family Dermatology, National City, USA

**Keywords:** action, doxycycline, drug, essential, hand, kinetic, monohydrate, medication, resting, tremor

## Abstract

There are several etiologies for acquired tremors. Medications have been observed to induce tremors. A 64-year-old man initiated oral doxycycline for scalp folliculitis. By the third dose, he noticed development of hand tremors. He continued the medication and the tremor persisted. Doxycycline was stopped after five days. Within three days of discontinuing the drug, all tremors had resolved. Medication-induced tremors have been associated with several drugs. These include antiarrhythmics, antibiotics, antidepressants, antiepileptics, antimycotics, antivirals, bronchodilators, chemotherapeutics, dopamine depleters, drugs of misuse, gastrointestinal drugs, hormones, immunosuppressants, methylxanthines, mood stabilizers, and neuroleptics. Several antibiotics have also been associated with drug-induced tremors. These include aminoglycosides, carbapenems, cephalosporins, fluoroquinolones, folate synthesis inhibitor, glycopeptides, macrolides, penicillins, and tetracyclines. Doxycycline can be added to the list of drugs associated with medication-induced tremors.

## Introduction

Tremors are classified as action (postural and kinetic) and resting. Tremor syndromes are further classified as cerebellar, drug-induced, dystonic, essential, Holme’s, palatal, Parkinsonian, peripheral, physiologic, and psychogenic. Several drugs have been associated with medication-induced tremors. Indeed, antibiotic treatment may result in drug-associated tremors [1,2].

Doxycycline is a bacteriostatic tetracycline antibiotic. It inhibits bacterial protein synthesis by reversibly binding to the 30S ribosomal subunit of the bacteria. This results in the prevention of aminoacyl transfer RNA (tRNA) binding to messenger RNA (mRNA) [3].

A man developed hand tremors after initiating treatment with doxycycline monohydrate. The tremors resolved after discontinuation of the drug. Antibiotics that have been observed to cause tremors are reviewed.

## Case presentation

A 64-year-old man presented for evaluation of scalp lesions that he had been scratching. His past medical history was significant for kidney transplants in 2008 and in 2016. He had no history of Parkinsonism. His immunosuppressive therapy following the first transplant included tacrolimus; he developed tremors which completely resolved once the tacrolimus was discontinued and did not occur after cyclosporine was initiated. His current medications include aspirin, carvedilol, cyclosporine, mycophenolate, nifedipine, and prednisone. 

Examination of his scalp showed crusted papules and erosions. A bacterial culture grew methicillin-susceptible *Staphylococcus aureus*. The organism was also susceptible to clindamycin, tetracycline, and trimethoprim-sulfamethoxazole.

Treatment was initiated with oral doxycycline monohydrate 100 mg twice daily. Following the third dose of doxycycline on the second day of treatment, he noted that his hands would shake. Specifically, after the morning dose they would shake until the mid-afternoon. He would take the evening dose and when he woke up in the morning, his hands were shaking.

He continued to take the doxycycline for five days and the symptoms persisted. He then discontinued the medication. Within three days after stopping the drug, his hand tremors had completely resolved.

When he returned for evaluation, two weeks after his original visit, there were still some residual scalp erosions. Cephalexin, at a reduced dosage of 250 mg twice daily, was initiated for ten days. Topical agents for his scalp were also prescribed: chlorhexidine 4% liquid for use in the shower each day and clindamycin 1% solution twice daily.

On follow-up examination two weeks later, his scalp folliculitis demonstrated resolution. His tremor had not recurred. Correlation of the clinical history, including initiation of symptoms after starting doxycycline and resolution after discontinuing the drug, established the diagnosis of medication (doxycycline)-induced hand tremors.

## Discussion

Medication-induced tremors have been observed following the initiation of several drugs. Commonly associated drugs include the following classes: antiarrhythmics, antibiotics, antidepressants, antiepileptics, antimycotics, antivirals, bronchodilators, chemotherapeutics, dopamine depleters, drugs of misuse, gastrointestinal drugs, hormones, immunosuppressants, methylxanthines, mood stabilizers, and neuroleptics [2]. Indeed, our patient experienced tremors after receiving tacrolimus; they resolved after stopping the drug and did not recur with cyclosporine.

Antibiotic-induced tremors are less frequently observed (Table 1) [4-16]. They are not restricted to a specific class of drugs and have occurred after initiating treatment with aminoglycosides, carbapenems, cephalosporins, fluoroquinolones, folate synthesis inhibitor, glycopeptides, macrolides, penicillins, and tetracyclines. To the best of our knowledge, tremors attributed only to doxycycline monohydrate have not previously been described.

**Table 1:** Antibiotics associated with drug-induced tremor

Class of drug	Specific medication	Reference
Aminoglycoside	Gentamicin	[4]
Carbapenem	Imipenem/cilastin	[5]
Cephalosporin	Cefuroxime	[6]
Fluoroquinolone	Ciprofloxacin, levofloxacin, trovafloxacin	[7-9]
Folate synthesis inhibitor	Trimethoprim-sulfamethoxazole	[10-11]
Glycopeptide	Vancomycin	[12]
Macrolide	Erythromycin	[13]
Penicillin	Carbenicillin, piperacillin / tazobactam	[14, 15]
Tetracycline	Doxycycline	Current report

The aminoglycoside gentamicin has been described to cause shaking in patients. A 21-year-old woman was treated for endometritis two days after a cesarean section. One hour after a gentamicin infusion, she experienced shaking, chills, and rigors that resolved with administration of intravenous diphenhydramine [4].

Imipenem/cilastin, a combination carbapenem medication, was observed to cause tremors. A 79-year-old man with renal failure presented with peritonitis; he was treated with intravenous imipenem/cilastin 500 mg every 12 hours. After three days, the patient was found to have a flapping hand tremor and an episode of absence followed by confusion. Imipenem/cilastin was discontinued and the tremor resolved within one week [5].

Cefuroxime, a cephalosporin, also caused tremor. A 61-year-old woman was admitted to the hospital for myoclonus, full-body tremors, and decreased consciousness that occurred after cefuroxime treatment. Neurologic symptoms resolved with hemofiltration and reduction of plasma concentration of cefuroxime [6].

Multiple fluoroquinolones have been associated with antibiotic-induced tremors. An 84-year-old patient who was treated with ciprofloxacin developed a palatal tremor and rhythmical movements of the face and trunk.

Both the tremor and movements resolved two days after discontinuing the antibiotic [7]. Neurologic adverse events, including tremor, developed in a 67-year-old man with an upper respiratory infection, who received oral levofloxacin and fulfenamic acid (a nonsteroidal anti-inflammatory drug). Four days later, he developed a chorea-like involuntary hand tremor, gait disturbance, and visual disturbances; seven days after initiation of levofloxacin, the patient experienced generalized convulsions. All neurologic symptoms resolved within a week of stopping both medications [8].

Trovafloxacin-induced tremors occurred in an 86-year-old woman with diabetes. She was treated for foot osteomyelitis with trovafloxacin and developed tremors in all extremities after five days. The tremors stopped after trovafloxacin was discontinued [9].

Trimethoprim-sulfamethoxazole has also been observed in multiple patients to cause tremor. A 66-year-old man with spinal cord injury and neurogenic bladder was treated with twice-daily trimethoprim-sulfamethoxazole. After three days, the patient noticed myoclonus and postural tremor of the hands and asterixis that resolved after the last dose [10].

There is also an association with tremor development in patients with acquired immunodeficiency syndrome (AIDS) who receive trimethoprim-sulfamethoxazole. A 46-year-old man with AIDS received the antibiotic for *Pneumocystis pneumonia*. He developed a high-frequency postural tremor of the head and all extremities [11].

There have also been reports of vancomycin-induced tremors. A 69-year-old woman with essential tremor was found to have prosthetic valve endocarditis. She was treated with a one-gram intravenous infusion of vancomycin three times daily. Two weeks after initiation, she developed severe whole-body tremors that were distinct from the patient’s underlying essential tremor. The tremor resolved thirty minutes after stopping the infusion [12]. 

The macrolide erythromycin was reported to cause a tremor in a 64-year-old man. One day after initiation of the antibiotic, he noted visual and auditory hallucinations and whole-body tremors that interfered with daily activity. The tremors were absent during sleep. All neurologic symptoms resolved within five days after cessation of therapy and did not recur [13].

The penicillin class of antibiotics has also been associated with medication-induced tremors. A 57-year-old woman with end-stage renal disease received piperacillin/tazobactam four times daily for bronchiectasis and pulmonary infection. After the fifth dose, she developed a tremor and two episodes of generalized tonic-clonic seizures that resolved with lorazepam and phenytoin. When a third seizure occurred after the sixth dose of piperacillin/tazobactam, the patient was switched to ciprofloxacin and did not have reoccurrence of tremors or seizures [14].

Carbenicillin-induced tremors have also been observed. A 15-year-old boy with acute lymphoblastic leukemia was found to be neutropenic and febrile after initiation of induction chemotherapy. He was started on gentamicin three times daily and carbenicillin four times daily. One day later, he developed tremors of extended arms that progressed to involve the whole body within 24 hours. Due to persistence of fever, antibiotic therapy was switched to ceftazidime and gentamicin; all neurologic symptoms resolved within two days. On a second episode of neutropenic fever four weeks later, he again received gentamicin and carbenicillin; tremors recurred 12 hours after initiation and again resolved when switched to ceftazidime [15].

Serotonin syndrome is another sequalae of medication administration that can lead to tremor. It presents with autonomic instability, behavioral changes, and neurologic symptoms such as tremor, myoclonus, and rigidity [16]. Multiple antibiotics have been observed to cause serotonin syndrome by interacting with antidepressant medications (Table 2) [16-18]. These antibiotics include fluoroquinolones (ciprofloxacin), folate-synthesis inhibitors (trimethoprim-sulfamethoxazole), and oxazolidinones (linezolid).

**Table 2:** Antibiotics associated with antidepressant-induced serotonin syndrome

Class of drug	Specific medication	Reference
Fluoroquinolones	Ciprofloxacin	[17]
Folate synthesis inhibitor	Trimethoprim-sulfamethoxazole	[18]
Oxazolidinone	Linezolid	[16]

A 61-year-old man who had major depressive disorder that was being treated with venlafaxine presented to the hospital with intention tremor, myoclonus, hyperreflexia, and fever after receiving ciprofloxacin. After three days of tapering venlafaxine and discontinuation of ciprofloxacin, the patient’s symptoms resolved [17].

A 46-year-old woman with venlafaxine-treated depression received trimethoprim-sulfamethoxazole twice daily for cystitis and developed a severe resting hand tremor after four days. Serum concentration of venlafaxine was increased from baseline and a drug interaction between trimethoprim-sulfamethoxazole and venlafaxine was believed to be the cause. After stopping the antibiotic, the tremor resolved [18].

An 82-year-old woman with depression who was being treated with sertraline was found to have a hospital-acquired infection. She was given linezolid; 12 hours later, she developed altered mental status, fever of 104.5 degrees Fahrenheit, and tremors and rigidity of the extremities. Serotonin syndrome in the setting of a sertraline and linezolid interaction was suspected and both therapies were halted. Neurologic symptoms resolved within two days [16].

Doxycycline-induced tremor has also, albeit rarely, occurred as a result of an interaction of the antibiotic with another medication. A 68-year-old man, with bipolar type 1 disorder treated with lithium, received doxycycline for bronchitis. His serum concentrations of lithium increased and he developed ataxia, disorientation, memory loss, and a severe tremor [19]. To the best of our knowledge, none of the medications our patient was receiving have been observed to interact with doxycycline and result in tremors.

## Conclusions

Medication-induced tremors should be considered in patients with a new onset of tremors. The tremors can be generalized or restricted to specific body parts, such as the hands in our patient. Doxycycline monohydrate can be added to the list of antibiotics associated with medication-induced tremors.
